# Sub-microscopic analysis of t-tubule geometry in living cardiac ventricular myocytes using a shape-based analysis method

**DOI:** 10.1016/j.yjmcc.2017.05.003

**Published:** 2017-07

**Authors:** Cherrie H.T. Kong, Eva A. Rog-Zielinska, Clive H. Orchard, Peter Kohl, Mark B. Cannell

**Affiliations:** a School of Physiology, Pharmacology & Neuroscience, Faculty of Biomedical Sciences, University of Bristol, University Walk, Bristol BS8 1TD, United Kingdom; b National Heart and Lung Institute, Harefield Heart Science Centre, Imperial College London, Harefield UB9 6JH, United Kingdom; c Institute for Experimental Cardiovascular Medicine, University Heart Centre Freiburg – Bad Krozingen, Medical School of the University of Freiburg, Elsaesser Str 2Q, 79110 Freiburg, Germany

**Keywords:** E-C, excitation-contraction, EM, electron microscopy, ET, electron microscopic tomography, FWHM, full-width at half maximum, SS, surface sarcolemma, TTs, transverse-axial tubules, *D*_*TT*_, t-tubule diameter, *V:SA*, volume to surface area ratio, Confocal imaging, Image processing, Fluorescent dyes, Cardiac myocyte, T-tubules, Geometry

## Abstract

Transverse-axial tubules (TTs) are key structures involved in cardiac excitation-contraction coupling and can become deranged in disease. Although optical measurement of TTs is frequently employed to assess TT abundance and regularity, TT dimensions are generally below the diffraction limit of optical microscopy so determination of tubule size is problematic. TT diameter was measured by labeling both local surface membrane area and volume with fluorescent probes (FM4-64 and calcein, respectively), correcting image asymmetry by image processing and using the relationship between surface area and volume for a geometric primitive. This method shows that TTs have a mean (± SEM) diameter of 356 ± 18 nm in rabbit and 169 ± 15 nm in mouse (*p* < 0.001). Rabbit TT diameters were more variable than those of mouse (*p* < 0.01) and the smallest TT detected was 41 nm in mouse and the largest 695 nm in rabbit. These estimates are consistent with TT diameters derived from the more limited sampling of high-pressure frozen samples by electron tomography (which examines only a small fraction of the cell volume). Other measures of TT abundance and geometry (such as volume, membrane fractions and direction) were also derived. On the physiological time scale of E-C coupling (milliseconds), the average TT electrical space constant is ~ 175 μm in rabbit and ~ 120 μm in mouse and is ~ 50% of the steady-state space constant. This is sufficient to ensure reasonable electrical uniformity across normal cells. The image processing strategy and shape-based 3D approach to feature quantification is also generally applicable to other problems in quantification of sub-cellular anatomy.

## Highlights

• Living cardiomyocytes were dual-labeled with fluorescent surface and volume probes.
• A novel 3D image processing strategy enabled calculation of t-tubule diameter.
• The method shows rabbit and mouse t-tubules have quite different morphologies.
• Mean diameters of rabbit and mouse t-tubules were 360 and 170 nm, respectively.
• Estimated electrical space constants are sufficient to ensure electrical uniformity.

## 1 Introduction

In cardiac muscle, the transverse-axial tubules (TTs) are invaginations of the surface sarcolemma (SS) that form a complex network throughout the cell [1], [2]. TTs enable rapid propagation of the action potential throughout the cell, and permit near synchronous excitation-contraction (E-C) coupling in ventricular [3] and atrial cardiomyocytes [4] via activation of “calcium release units” [5]. Many disease states are associated with disruption of TT structures and, in heart failure and this correlates strongly with reduced contractile performance (e.g. [6], [7]). Among pathological changes in the TT system are local dilations, loss of t-tubules and their malformation [7], [8], [9], [10] that accompany the progression to heart failure. It has also been proposed that altered diffusion between the TT lumen and the extracellular space may take place [11].

Although previous studies have already quantified some features of TT organization in living cells, for example TT regularity (e.g. using Fourier analysis [7]), density [12], [13] and orientation [6], [8], few have measured TT diameter in living myocytes. The primary obstacle to direct optical measurement of TT diameter is the limitation imposed by the conventional optical diffraction limit. Electron micrographs suggest that mouse TT diameters are likely to be 40–200 nm [14], [15] which will be unresolved in confocal microscopes with a diffraction limit of ~ 200 nm. Super-resolution microscopy (such as Stimulated Emission Depletion, or STED microscopy) can reveal the lumen of some tubules [9], but such methods are technically demanding, have a more limited axial resolution and may not always be applicable to living cells (the latter being desirable to avoid fixation artifacts and to allow correlation with function). As a more accessible alternative, the limited optical resolution of a (confocal) microscope can be circumvented by immersing cells in a fluorescent medium and using signal intensity as an indicator for TT size [16], [17]. The latter methods rely on accurate segmentation to produce a binary skeleton of the TT network, which is needed to control for the complexity of TT branching, and this becomes more difficult at smaller TT diameters (due to decreasing signal-to-noise ratio).

The mouse is a widely used model for studying cardiac function. However, existing data suggests that murine cardiac TTs are likely to be narrower, and therefore harder to image, than those of human [8] and other laboratory animals, such as rat (~ 0.25 μm diameter [16]) or rabbit (~ 0.45 μm diameter [17]) (see also Discussion). We therefore developed a new dual wavelength, intensity based 3D analysis method to quantify local TT geometry. We have applied this method to confocal images of mice and rabbit ventricular cardiomyocytes (two commonly used small animal models). Simultaneous imaging of two dyes (calcein and FM4-64) provides measures of local TT volume and surface area from which we can then derive, using a cylindrical model, local TT diameter and geometry. This improves the reliability of TT detection/characterization, as well as providing a cell-wide measure of TT abundance. The utility of the method for deriving a quantitative measure of TT width was confirmed using ultra-rapid high pressure cryo-fixation and 3D electron tomography reconstructions after freeze substitution [18].

## 2 Materials and methods

### 2.1 Myocyte preparation

All procedures were performed in accordance with the Animals (Scientific Procedures) Act (UK). Ventricular myocytes were enzymatically isolated from the hearts of male mice (C57BL/6, 25 g) or rabbits (New Zealand White, 2.5 kg), as described previously [19], [20]. Briefly, rabbit and mouse hearts were rapidly removed and washed in a standard physiological saline solution (see below) that contained 0.1 mM CaCl_2_ and 10 IU/mL heparin. The aorta was mounted on a Langendorff system (37 °C) for perfusion with oxygenated standard solution for 5 min, followed by standard solution containing 0.1 mM CaCl_2_, 0.8 mg/mL collagenase II (Worthington Corp., USA) and 0.6 mg/mL protease XIV (Sigma-Aldrich Co. Ltd., U.K.) for 15 min. The ventricles were then minced and filtered to isolate single cells. The cells were centrifuged and re-suspended in storage solution (see below).

### 2.2 Solutions

The standard solution used for mouse cell isolations contained (in mmol/L): 130 NaCl, 5.4 KCl, 1.4 MgCl_2_, 0.4 NaH_2_PO_4_, 10 d-glucose, 4.2 HEPES, 20 taurine and 10 creatine, pH = 7.4. For rabbit cells, the solution was similar, except that it contained 4.5 KCl, 3.5 MgCl_2_ and 5 HEPES. The cell storage solution was a low Ca Kraftbrühe (KB) medium to relax cells and prevent possible TT compression by cell contraction, which contained: 100 L-glutamic acid, 30 KCl, 10 HEPES, 1 EGTA, 5 Na pyruvate, 20 taurine, 20 glucose, 5 MgCl_2_, 5 succinic acid, 5 creatine, 2 Na_2_ATP, 5 ß-OH butyric acid. All experiments were performed at room temperature in KB medium.

### 2.3 Confocal imaging

Cell membranes were labeled with 5 μM FM4-64 (Thermo Fisher, USA) for 5 min, washed, then bathed in 400 μM calcein as an extracellular marker (Sigma-Aldrich). This dye selection allowed good separation of fluorescence signals, although other dye pairs could be used with suitable correction of cross-talk between channels if necessary. Cells were imaged using a LSM 880 (Carl Zeiss, Germany) with an Airyscan detector set to super-resolution mode, and water-immersion objective with 40 × magnification and 1.2 numerical aperture. Dyes were excited with the 488 nm Argon laser line, and fluorescence recorded at 495–550 (calcein) and > 605 nm (FM4-64). Volume images were recorded at 12-bit resolution with voxel size set to 60 nm in the focal plane (x-y) and ~ 180 nm along the optical axis (z) to ensure oversampling.

### 2.4 Measurement of TT width in living myocytes

A novel method was developed to assess TT width in living myocytes. The algorithm was written in MATLAB R2015a (MathWorks, Inc., USA), and ImageJ (v1.50f, National Institutes of Health, USA) was used to aid the analysis. (Codes can be obtained by contacting the authors.)

The improved method relies on microscopic measurement of the local TT luminal volume (calcein signal) and membrane area (FM4-64 signal) to calculate the volume to surface area ratio, *V:SA*. For any given shape *V:SA* increases with size, so *V:SA* can be used to compare object sizes with no explicit assumptions beyond shape similarity. *V:SA* can be calibrated to physical dimensions using a geometric model, as described below.

**Fig. S1:**
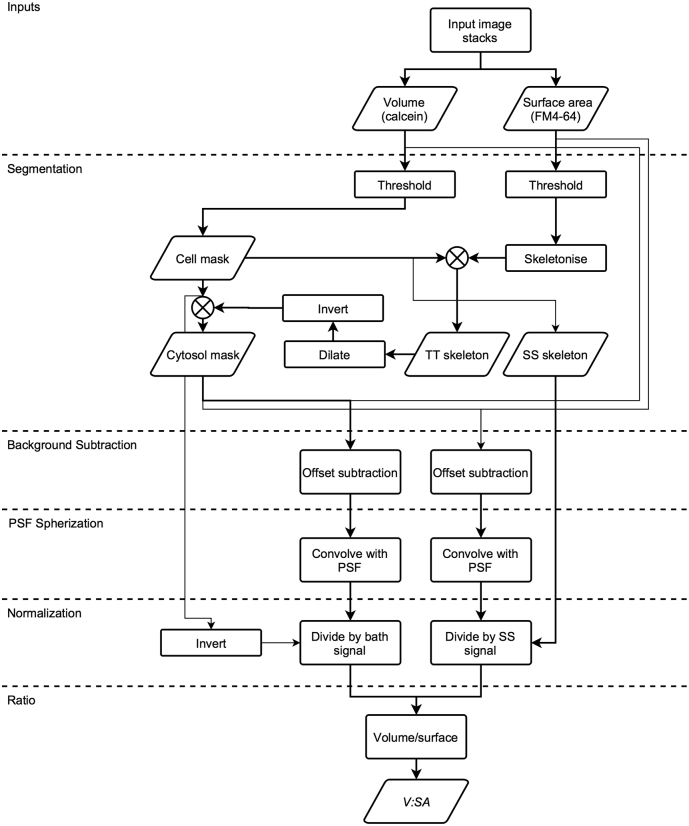
Flow diagram of the *V:SA* method for determining TT diameter. For additional details, see Materials and methods (Section 2.4). The algorithm used input data provided by stacked confocal images of two signals: a membrane signal (e.g. FM4-64), and an extracellular space signal (e.g. calcein). The data is then used to create masks that identify the structures of interest, namely, the cell, the cytosol, the t-tubules (TTs) and the surface sarcolemma (SS). Using these masks, the data is background-subtracted and blurred to produce a symmetric PSF. This is followed by normalization so that the volume and surface membrane signals are 1.0 in both the bath and at the SS. The volume signal is then divided by the surface membrane signal to produce the *V:SA* image. The *V:SA* is positively-related to TT width for a given shape.

The ratio of the calcein to FM4-64 signal intensities is a measure of *V:SA*. Since *V:SA* increases linearly with t-tubule diameter (*D*_*TT*_), it can be used to monitor changes in average *D*_*TT*_. As pointed out previously, use of confocal fluorescence intensity signals assumes a spatially invariant and symmetric probe point spread function (PSF) [16]. This can be realized by suitably blurring the data produced by an asymmetric or aberrated confocal PSF. Fig. S1 illustrates the *V:SA* method with a flow diagram showing the image processing steps. Briefly, calculation of the TT *V:SA* involved segmentation of cell interior and surface, background subtraction, conversion of the normal microscope asymmetric PSF to a spherical PSF, and data normalization to remove experimental variations in dye concentration and to permit calibration to size units.

A TT skeleton was produced by applying an Otsu threshold to produce a binary image mask, which was then skeletonized by thinning (via an ImageJ plugin based on [21]) until objects were reduced to single-pixel wide lines in 3D. A cell area mask was created by applying a threshold to the calcein signal and applying a morphological closing operator to remove any artifactual ‘holes’ in the mask. The cell border was refined from the local gradients of both signals, so that only in-focus and non-z-groove regions were used as the surface sarcolemma (SS skeleton). A TT skeleton was created by combining the cell area mask and membrane skeletons.

For both signals, background subtraction was achieved using pixels within the cytosol mask outside the TT skeleton. The background values for TT pixels were nearest-neighbor interpolated from surrounding pixels in the cytosol mask. This background image was then subtracted from the data.

To overcome the problem of an asymmetric confocal PSF, which would cause signal intensity to depend not only on TT width, but also TT orientation [16], the data was blurred to reduce the x-y resolution to that of the z-resolution, making the effective PSF spherical. This involved convolution with a 3D Gaussian function whose x-y dimensions were matched the z-resolution of the measured PSF (obtained from images of 0.17 μm Yellow-Green Fluorospheres, Thermo Fisher).

To control for variations in dye concentration and microscope efficiency, the calcein (volume) signal was normalized to that recorded from in the perfusion bath, and the FM4-64 (surface membrane) signal to that at the cell surface sarcolemma (SS). *V:SA* images were calculated by division of these normalized images. Thus, *V:SA* at the SS should be ~ 0.5, while the *V:SA* at the TT skeleton will be proportional to *D*_*TT*_ according to

(1) $$V:\mathrm{SA}=\frac{D_{\mathrm{TT}}}{2\mathrm{FWHM}}∙\sqrt{\frac{\ln\left(2\right)}{\pi }}$$

where FWHM is the full-width at half maximum of the modified microscope PSF (see Theory in Supplementary materials).

### 2.5 Electron microscopy/tomography and analysis

TT shape and dimensions were also measured with 3D electron tomography (ET) and electron microscopy (EM), for comparison with our optical method. Isolated cells were re-suspended in the standard physiological solution to which 10% BSA was added, left to sediment into a pellet, and then high-pressure frozen [18] with an EM PACT2 + RTS High Pressure Freezer (Leica Microsystems, Germany) with liquid N_2_ at 2000 bar. Frozen samples were freeze-substituted in 1% osmium tetroxide and 0.1% uranyl acetate (in acetone), and subsequently embedded in Epon resin. All sections were stained with uranyl acetate and lead citrate; sections for tomography were additionally coated with 15 nm colloidal gold particles. Sections (80 nm) for EM were analyzed using 100 kV Tecnai 12 transmission electron microscope (FEI Company, USA) fitted with a TVIPS F214 digital camera. Sections (380 nm) for ET were analyzed using 200 kV Tecnai T20 transmission electron microscope fitted with an Eagle 4k × 4k camera (FEI Company). For ET, images were acquired in a double tilt series (with 90° sample rotation between series), between ± 70° using the Saxton tilt increment scheme [22]. Tilt series were aligned, reconstructed and combined using IMOD software [23], [24].

### 2.6 Further geometric analysis of TT network

The FM4-64 signal was also used to quantify some features of TT organization. TT length was obtained from the 3D skeleton (see above), while tubule orientation was calculated from the Eigenvector of the image [17]. Tubule orientation was defined with respect to the cross-section of the cell, so that tubules oriented at 0 ± 15° from this transverse plane were referred to as “transverse”, and tubules oriented at 90 ± 15° were considered “axial”.

### 2.7 Data presentation

Mean data is presented with one standard error of the mean (SEM), unless otherwise stated. Statistical testing was performed with Prism v6.01 (Graphpad, USA), using the Mann-Whitney Test, or Spearman rank correlation, unless otherwise stated. Statistical significance * was defined by *p* < 0.05, and ** is used to indicate *p* < 0.001.

## 3 Results

### 3.1 Measurement of TT diameter in living rabbit myocytes

**Fig. 1:**
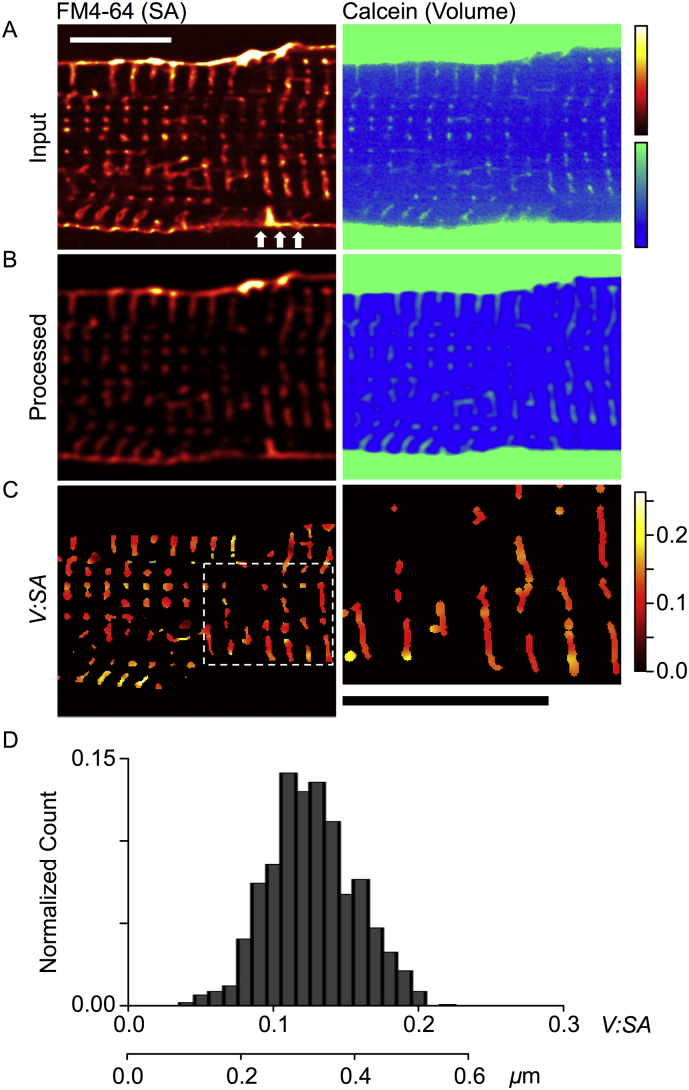
Application of the *V:SA* method to TTs in rabbit ventricular myocytes. (A) Shows a region of a myocyte dual labeled with FM4-64 for the surface membrane (left panel), and calcein for the extracellular space -which includes the TT lumen (right panel). (B) Shows the same optical sections after processing (background subtraction, PSF modification to a sphere and normalization). (C) The left panel shows the *V:SA* values calculated for pixels within the TT mask. The right panel shows a magnified view of the region marked in C (dashed white box), where the TT skeleton was dilated to reflect the calculated TT width and the color table indicates *V:SA*. Scale bars show 10 μm. (D) A histogram of *V:SA* values within the TT skeleton from the cell shown in A-C. The lower scale bar shows conversion from *V:SA* to TT diameter. Means of all data are given in Table 1.

**Fig. S2:**
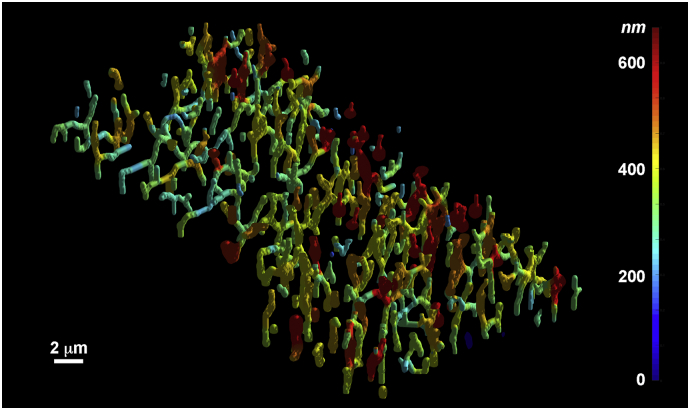
3D rendered overview of rabbit TTs. The surface color corresponds to TT diameter (nm), as indicated on the color bar at right. Spatial scale bar 2 μm (2000 nm). Note the presence of local TT dilations along a single tubule. Some of these TTs would appear as single objects within a single 2D confocal plane of section.

Rabbit ventricular myocytes, which are known to have wider TTs than mice, were used to assess the utility and accuracy of the *V:SA* method to determine TT width in living myocytes. Fig. 1A shows a region of a myocyte with the surface membrane labeled with FM4-64 (left panel), and extracellular space with calcein (right panel). There is a remarkably close association between the two dye signals. A Spearman rank correlation of pixel intensities within the TT mask supports this observation (rho, of 0.99; *p* < 0.001). Closer inspection reveals that some TT invaginate from the SS at grooves (arrowed), confirming that FM4-64 labels the TT membrane, while calcein labels the TT lumen (which is continuous with the extracellular space). Punctate labeling, closer to the cell center corresponds to TTs running orthogonal to the image plane, as seen in 3D data sets (Fig. S2). Fig. 1B shows the same optical sections after background subtraction, PSF conversion to a sphere and normalization (see Methods, and Fig. S1). The division of the volume signal (right panel) by the surface area signal (left panel) results in the *V:SA* image (Fig. 1C, left panel), which has had a TT mask applied for display purposes (since cytosolic pixels include divisions by zero). The right panel shows an enlarged region of Fig. 1C (indicated by dashed rectangle), illustrating the spatial variations in TT width that probably correspond to local TT dilations as previously described [16], [17], [25]. Fig. 1D shows the distribution of calculated *V:SA* in the same imaging plane, with a mean of 0.123 ± 0.022 (SD, for *n* = 891 pixels). The group mean *V:SA* (*n* = 11 cells, 3 rabbits) was 0.139 ± 0.007 (SEM).

**Fig. 2:**
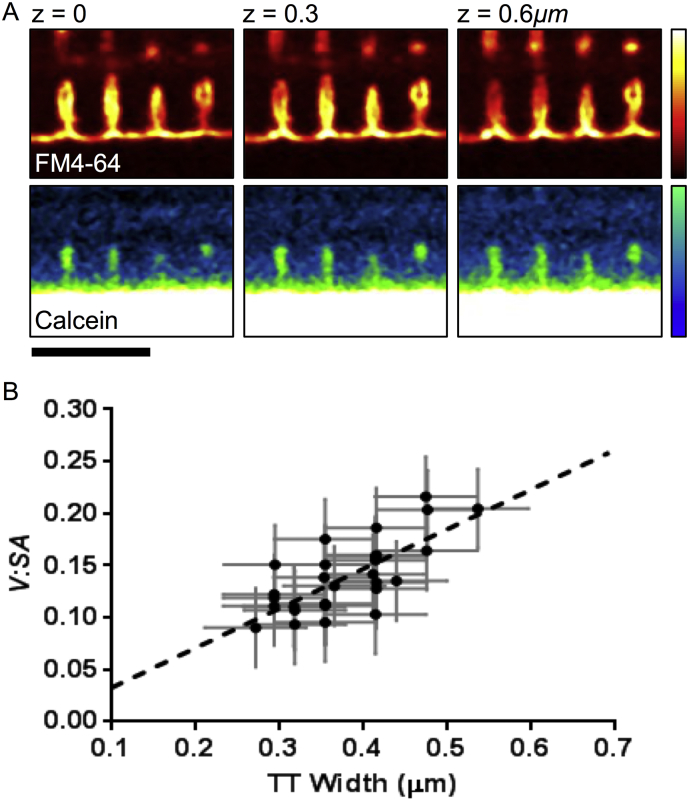
Estimation of TT diameter from the *V:SA* ratio. (A) Exemplar rabbit TTs with diameters above the optical resolution limit (i.e. showing a visible lumen). Position of the imaged plane along the optical axis, z, is indicated. The top panels show FM4-64 staining, highlighting the TT membranes invaginating from the SS z-grooves. Evidence showing that the space between the TT membranes is the lumen of a single TT is provided by the calcein dye signal. Scale bar 5 μm. (C) Tubule width measured manually, compared to the measured *V:SA* (filled circles, *n* = 30 tubules). The dashed line shows linear regression, which has a slope of 0.38 ± 0.07, which is not significantly different to that expected from a cylindrical model of TTs (0.39, see Supplementary Materials, Eq. 7).

Using a cylindrical model of TT shape (see Methods), this *V:SA* corresponds to a TT diameter of 0.356 ± 0.018 μm in rabbit ventricular myocytes (Table 1). The validity of using a simple cylindrical model was assessed by comparing directly measured TT widths and those calculated using *V:SA* values. Since rabbit TTs are relatively large, some TTs are above the limit of optical resolution of our microscope. Examples of such tubules are shown in Fig. 2B, where optical slices at the indicated z values are shown. In places, grooves along the SS invaginated to become TTs that proceed into the next imaging plane. The notion that the decrease in signal between two adjacent TT membranes may represent a TT lumen (rather than a thin tubule splitting) is supported by the calcein signal, which shows that extracellular labeling is centered in the region of a FM4-64 signal void that would correspond to a TT lumen. There was good agreement between estimated *D*_*TT*_ from the cylindrical model and measured widths (Fig. 2B, solid circles). At least part of the scatter in the relationship arises from the pixelation of the data, with a resolution of 0.06 μm (as indicated by the horizontal error bars). The signal to noise ratio in *V:SA* signal was typically ~ 13 and this increased the statistical uncertainty in the empirical relationship between observed and *V:SA*-derived diameters. Nevertheless, the agreement between observed and predicted relationship was significant, with linear regression (Fig. 2B, dashed line) of the measured data yielding a slope of 0.38 ± 0.07. This was not different to that predicted from the circular geometric model (0.39; *p* = 0.88, ANOVA) and shows that the low molecular weight volume dye was not physically excluded from the TT lumen.

**Table 1:** Parameters of TT network as measured and/or calculated (italicized) from *V:SA* datasets. Numbers give mean ± SEM. Mann-Whitney test for significance is shown as ** where *p* < 0.001.

Parameter	Rabbit	Mouse
n (cells/hearts)	11/3	12/3
V:SA	0.139 ± 0.007	0.066 ± 0.006**
*Diameter, D*_*TT*_*(μm)*	0.356 ± 0.018	0.169 ± 0.015**
Skeleton length (μm/μm^3^)	0.27 ± 0.02	0.70 ± 0.04**
Transverse tubules (%)	63 ± 4	37 ± 1**
*TT membrane abundance (μm*^*2*^*/μm*^*3*^*)*	0.30	0.37
*TT volume as % cell volume*	2.5	1.6
*% Cell membrane in TT*	65	51

### 3.2 TT diameter in mouse ventricular myocytes

**Fig. 3:**
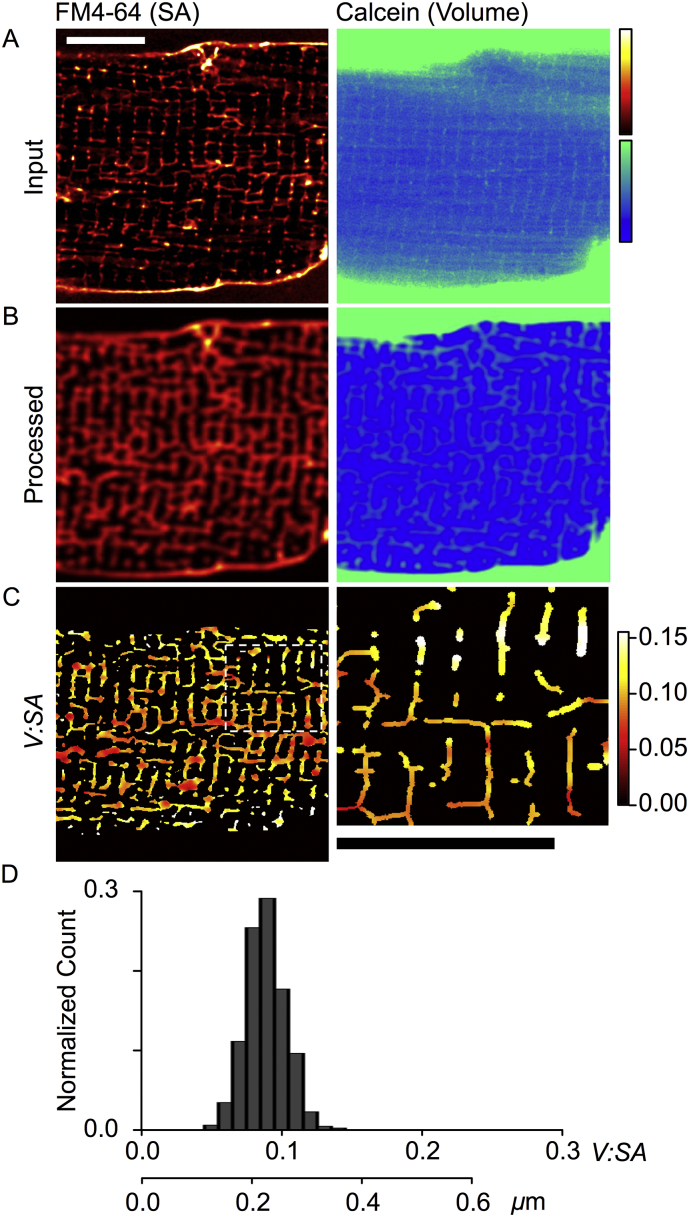
Application of the *V:SA* method to TTs in mouse ventricular myocytes. (A) Shows a region of a myocyte co-labeled with FM4-64 for the surface membranes (left panel), and calcein for the extracellular space and TT lumen (right panel). (B) Shows the same optical sections after image processing as described for Fig. 1B. (C) The left panel shows the *V:SA* values calculated for pixels within the intermediary TT mask. The right panel shows the region marked in C (dashed white box). Scale bars show 10 μm. (D) Shows histograms of *V:SA* values within the TT skeleton from the cell shown in A-C. The lower scale bar shows conversion from *V:SA* to TT diameter. Mean data is given in Table 1.

Application of the *V:SA* method to analyze TTs in mouse ventricular myocytes is shown in Fig. 3. The input (Fig. 3A) and processed data (Fig. 3B) illustrate the markedly increased complexity of the mouse TT system, compared to rabbit TTs (e.g. compare to Fig. 1A and B). This includes a greater density of TTs per cell volume and a greater propensity for tubules to have an axial orientation. Visual examination of the calcein signal (which has been scaled so that TTs are visible at the expense of a saturated bath signal) shows that the TT signal is smaller and closer to background, implying that local TT volumes are smaller than in the rabbit. The calculated *V:SA* (Fig. 3C) was generally smaller than that for rabbit (Fig. 1C), supporting the visual impression. Fig. 3D shows a histogram of *V:SA* values for a representative murine cardiomyocyte, where the mean *V:SA* was 0.089 ± 0.015 (SD, for *n* = 2057 pixels). The group mean *V:SA* (for *n* = 12 cells, 3 mice) was 0.066 ± 0.006 (SEM), indicating that *D*_*TT*_ in mouse ventricular myocytes is approximately half that seen in the rabbit (*p* < 0.001). Using our cylindrical calibration, this *V:SA* corresponds to a mean *D*_*TT*_ of 0.169 ± 0.015 μm (Table 1). It is also apparent that mouse TT diameter was less variable than seen in rabbits (F = 5.48, *p* < 0.01).

### 3.3 Comparison of optical assessment of TTs diameters with EM and ET data

**Fig. 4:**
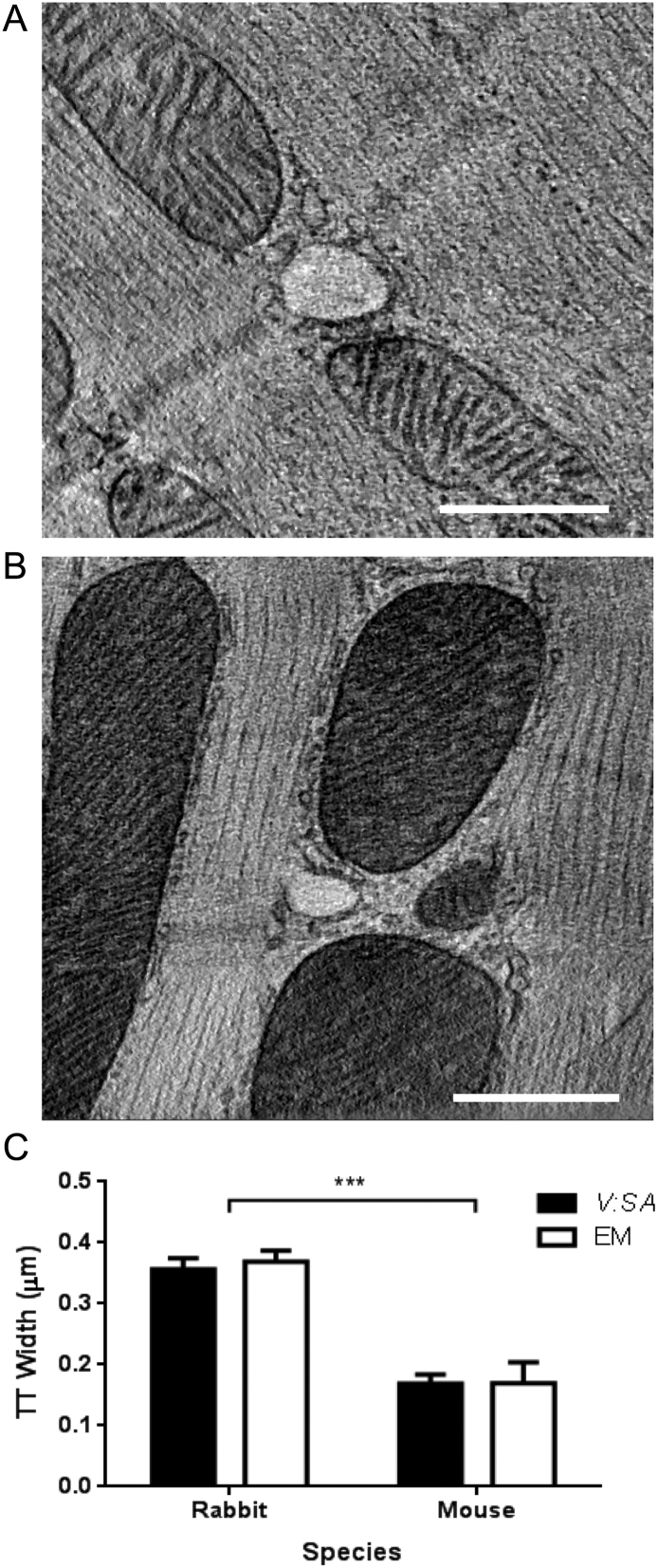
Representative electron tomography slices of TTs in isolated ventricular myocytes from (A) rabbit, or (B) mouse. Scale bars show 500 nm. (C) Measurements of TT width using the *V:SA* method and from EM from rabbit and mouse myocytes. Mouse TTs were ~ 2-fold narrower than rabbit TTs (*p* < 0.001).

Fig. 4A and B show exemplar images of a TT cross-section from rabbit, and from mouse, reconstructed in a TT-orthogonal plane (true cross-section) from 3D ET data [26]. The mean *D*_*TT*_ in rabbit cardiomyocytes (0.339 ± 0.021 μm, *n* = 29) was twice as large as that in mouse cells (0.175 ± 0.011 μm, *n* = 12, *p* < 0.001) (Fig. 4C). These absolute values and the species-related differences are in good agreement with those obtained by the optical method presented here. With ET, the ellipticity of the tubules can also be determined and this was 0.74 ± 0.02 (*n* = 12) in mouse and 0.75 ± 0.01 (*n* = 29) in rabbit. These values were not different between species (*p* > 0.62).

### 3.4 Further analysis of the TT network

The optical data used for the *V:SA* method also provides information about the organization of the TT network, including TT density and TT orientation, both of which have been shown to have important implications for E-C coupling. TT length per cell volume, determined from the FM4-64 signal, was 0.70 ± 0.04 μm/μm^3^ in murine cardiomyocytes. Further segmentation of the TT network using Eigenvectors enabled us to identify transverse and axial components (note that oblique elements outside a 15° cone centered around the axial or transversal direction, were excluded). This analysis revealed that transverse tubules comprised ~ 37% and axial tubules ~ 11% of total tubules in mice.

TT abundance in rabbit myocytes was 39% of that in mouse (rabbit: 0.27 ± 0.02 μm/μm^3^, mouse: 0.70 ± 0.04 μm/μm^3^, *p* < 0.001, Table 1). The proportion of transverse tubules was ~ 63%, and axial tubules were ~ 3%, supporting the visual impression that rabbit cardiomyocytes have fewer axial TTs (see Figs. 1A and 2A). It should be noted that such a wide area survey is difficult with EM, but straightforward with optical microscopy as shown here.

## 4 Discussion

### 4.1 Application of the *V:SA* method to ventricular myocytes

By using two spectrally-separable fluorescent dyes to simultaneously label TT membrane and TT lumen, the volume to surface area ratio (*V:SA*) can be calculated and compared across groups (e.g. rabbit vs. mouse as shown here). In addition, we show how the *V:SA* may be converted to physical dimensions, using a simple, reasonable geometric model of TTs. Our *V:SA* method revealed that rabbit TTs were generally twice as wide as those of mice, with mean *D*_*TT*_ of ~ 0.36 and ~ 0.17 μm, respectively (Fig. 4C, Table 1). These values were in remarkable agreement with those measured using ET of high-pressure frozen cells from the same species (Fig. 4C). High pressure freezing was chosen, as it avoids shrinkage artifacts associated with chemical fixation [18].

### 4.2 Use of a cylinder as model for TTs

By using ET, we avoid the problem of the plane of section making TTs appear to be elliptical even if they have circular cross-sections. Despite the almost 2-fold difference in rabbit and mouse TT diameter (Fig. 4) both had a similar degree of ellipticity (*E* = 0.75). As *E* decreases from 1.0 (a circle), *V:SA* decreases for a given width. However, this effect is small at *E* > *0.6*. For example, at *E* = 0.75 for the same *V:SA* value in rabbit (Fig. 1D) TT width would have been 0.40 μm and 0.32 μm along the major and minor axes, respectively, compared to 0.36 μm for a circular cross-section. It should be noted that these results should not suffer from uncertainty arising from the inevitable sample shrinkage associated with conventional fixation methods. A previous study on living cells also suggested that rabbit TTs may be elliptical in cross-section (mean Eigenvalue ratio *E* = 0.73) in reasonable agreement with our ET measurements [17]. In contrast to these results, a recent STED microscopy study in mouse [9] showed almost no ellipticity in TT dimensions in mouse, but we note that the mean TT diameter in that study (0.198 μm) was somewhat larger than measured here (see below).

### 4.3 TT diameter and length in mice

In murine ventricular myocytes, an early study using chemical fixation and subsequent stereological analysis of EM images reported *D*_*TT*_ of 0.05–0.12 μm [15], while a more recent study using super-resolution fluorescence microscopy, using paraformaldehyde-fixed cells reported *D*_*TT*_ of 0.24–0.28 μm [25]. Another super-resolution (STED) microscopy study suggested that the mean *D*_*TT*_ was ~ 0.2 μm [9]. This value is slightly larger than our estimate of ~ 0.17 μm, but TTs below the resolution of the STED microscope (~ 60 nm) would cause their estimate of the average *D*_*TT*_ to increase and a 50% contour measurement could also add a part of the PSF FWHM to the measure. With these effects in mind, we suggest that there is reasonable concordance with our measurements, but it should be noted that our method is faster and samples more tubules without explicit tubule-by-tubule selection and measurement.

In other mouse studies using EM, TT surface area per cell volume ranged from 0.17 to 0.55 μm^2^/μm^3^, and the percentage of cell volume occupied by TTs ranged from 0.8 to 3.2% [14], [27], [28]. From our data, using a mean *D*_*TT*_ of 0.17 μm (Fig. 4C, Table 1) and TT length 0.7 μm/μm^3^ (Table 1), the TT surface area and TT volume per cell volume would be 0.37 μm^2^/μm^3^ and 1.6%, respectively, values that are in the middle of the ranges derived from the aforementioned studies. The close concordance of these measures suggests that our method produces accurate estimates of TT geometry in living cells.

Simultaneous measurement of cell capacitance and volume suggests a total membrane area of 0.71 μm^2^/μm^3^ in mouse ventricular myocytes [29], implying (using 0.36 μm^2^/μm^3^ see Table 1) that mouse TTs could contribute up to ~ 51% of the total cell membrane area, which is within the range reported by EM (for summary see [28]). Recent electrophysiological studies in rat, using osmotic shock to disconnect ~ 84% of TTs, showed a decrease in mean cell capacitance from 260 pF to 179 pF [20]. Had all the tubules been disconnected, we can calculate that the average membrane capacitance should have decreased to ~ 164 pF, suggesting that disconnected TTs represent ~ 37% of total cell capacitance. This value can be converted to fractional area if the specific membrane capacity is known, but this depends on cholesterol mole fraction which may be quite different in TTs (see [30]). There is also uncertainty as to the extent/number of tubule mouths remaining after detubulation which would also lead to an underestimate of fractional TT area. Hence electrical detubulation experiments are likely to significantly underestimate fractional TT area and such electrical measurements may be consistent with TTs providing up to ~ 50% of total cell membrane area [30].

### 4.4 Measurements of TTs in rabbits

In rabbit, *D*_*TT*_ obtained by the *V:SA* method was also very similar to that measured using ET (Fig. 4C). Our measurements are slightly larger than those seen in EM data by Nelson and Benson [31], but somewhat smaller than those obtained by another live cell method [17], which suggested TT widths of ~ 0.45 μm. The former may have suffered from some chemical fixation-induced shrinkage while the latter was based upon applying a threshold to the intensity data, which can bias the size of a blurred object. In any case, if the mean *D*_*TT*_ were as large as 0.45 μm, then TT lumina should be visible in many more cases as this value is more than twice the diffraction limit of our microscope. Also, TTs would occupy a larger proportion of cardiac sarcomeres than is commonly seen. These considerations suggest that our method yields data that is compatible with the information currently available in the literature.

Previous EM studies of rabbit ventricular myocytes reported TT surface area per cell volume and fractional volume values of ~ 0.24 μm^2^/μm^3^ and 1.3–2.7%, respectively [28], [32], [33]. Our average *D*_*TT*_ of ~ 0.36 μm (Fig. 4C) and length of 0.27 μm/μm^3^ (Table 1) yields a TT surface area of 0.30 μm^2^/μm^3^, and a TT volume of ~ 2.7% of cell volume. Using the previously reported total surface membrane area to cell volume ratio of 0.46 μm^2^/μm^3^ in rabbit ventricular myocytes [34], we can estimate that TTs could comprise ~ 65% of total cell membrane area. This value may seem high, given the apparent sparcity of rabbit TTs. However, despite TT density being only ~ 39% of that in mice, the larger width of rabbit TTs results in a similar TT surface area per cell volume (0.30 μm^2^/μm^3^ in rabbit vs. 0.37 μm^2^/μm^3^ in mouse, Table 1).

### 4.5 TT space constant

The *D*_*TT*_ measurements presented here allows an estimate of the t-tubule space constant (*λ*_*TT*_) that determines electrical uniformity along t-tubules. From the well known steady state (dc) cable equation

(2) $$\lambda_{\mathrm{TT},\mathrm{dc}}=\sqrt{\frac{D_{\mathrm{TT}}R_{m}}{4R_{L}}}$$

and typical values of cardiac cell input resistance (45 MΩ) and cell capacitance (150 pF) [35] and a specific membrance capacitance of 1.0 μF/cm^2^ we estimate the membrane resistivity *R*_*m*_ to be ~ 7 kΩcm^2^. The lumen electrolyte resistance *R*_*L*_ is probably similar to that of physiological saline (~ 50 Ωcm [36]) suggesting an average *λ*_*TT*_ of ~ 350 μm and ~ 240 μm for rabbit and mouse respectively. However, the non-steady state response is frequency (*f*) dependent and the space constant is given by:

(3) $$\lambda_{\mathrm{TT},f}=\lambda_{\mathrm{TT},\mathrm{dc}}/R\left\{\sqrt{\left(1+i2\mathrm{\pi f}\tau_{m}\right)}\right\}$$

where τ_m_ is the membrane time constant. On the physiological (ms) time scale (corresponding to phases 0 and 1 of the action potential and *f* ~ 150 Hz), the space constant would decrease by ~ 50% to ~ 170 μm and ~ 120 μm for rabbit and mouse, respectively. This suggests that TT membrane potential should be quite uniform unless TTs become disconnected or damaged in some way so as to decrease the membrane input resistance.

### 4.6 Limitations and application to other cell systems

The current method cannot distinguish between two closely apposed fine t-tubules and a highly flattened larger t-tubule. Nevertheless, as a wide-scale analysis method it can give useful information by revealing possible differences between samples, even if the cause of the underlying difference may need to be explored by higher resolution methods. There is a potential for this general method to be employed in conjunction with a third, spectrally well-separated dye, to examine the relationship between TT geometry and local function (e.g. Ca release using a Ca indicator). Finally, the method is not limited to ventricular muscle but can be exploited wherever changes in surface area to volume ratios may be of interest. For example, changes in cable properties in neural circuits may also be due to changes in the diameter of dendrites/axons which could be amenable to the method shown here. In addition, estimates of the volume/size of synaptic (or other membrane) vesicles could also be obtained from consideration of the *V:SA* ratio of spheres.

## 5 Conclusions

By quantifying the signals which mark the surface and interior volume of subcellular structures, and applying a suitable geometric model, it is possible to measure the physical dimensions objects well below the conventional diffraction limit. This method is generally applicable to all confocal microscopes, and produces large volumes of quantitative data relatively simply. We have shown that the t-tubules of rabbits and mice are quite different in arrangement and size, with mean *D*_*TT*_ of ~ 360 nm and ~ 170 nm, respectively. Since it is known that there is extensive t-tubule remodeling in various disease states, the methods described here could provide powerful insight into subcellular remodeling of a key cellular structure.

The following are the supplementary data related to this article.

Supplementary material Image 1
