# Supplementary material for: Sub-microscopic analysis of t-tubule geometry in living cardiac ventricular myocytes using a shape-based analysis method
Source: J Mol Cell Cardiol. 2017 Jul;108:1–7. doi: 10.1016/j.yjmcc.2017.05.003 (PMC5529290; doi:10.1016/j.yjmcc.2017.05.003)
Supplement: Supplementary file 1 — Supplementary material [file mmc1.docx]

**Supplementary Materials and Methods**

**Theory Behind *V:SA* Method and Conversion to Physical Units**

A simple cylindrical model of TT shape was used to calculate the TT diameter (*D_TT_*) from *V:SA*. The local signal intensity, *SV_TT_* , of the volume dye for an unresolved, short cylindrical TT segment of length *δL*, is:

${SV}_{TT}=C_{Cal}\cdot\pi r^{2}\cdot\delta L$ Eqn. 1

where *C_Vol_* is a constant that depends on th concentration of the volume label (calcein) and microscope efficiency, and *r* is the radius of the cylinder fitting inside the local TT. Similarly, the local signal from the surface label, *SA_TT_* is given by:

${SA}_{TT}=C_{Area}\cdot2\pi r\cdot\delta L$ Eqn. 2

where *C_Area_* is concentration of the dye (FM4-64) inside the SS*. δL* is eliminated by division to give:

$\frac{{SV}_{TT}}{{SA}_{TT}}=\frac{C_{Vol}}{C_{Area}}\cdot\frac{r}{2}$ Eqn. 3

Eqn. 3 shows that the ratio of both signals at a TT depends on local radius which overcomes the uncertainty in the TT geometry beyond the assumption that all TT segments are reasonably represented by circular cross sections. As shown in the Results, this is a reasonable approximation but for any elliptical cross-section Eqn. 3 becomes:

$\frac{{SV}_{TT}}{{SA}_{TT}}=\frac{C_{Vol}}{C_{Area}}\cdot\frac{AE}{\left( 1+E \right)\left( 1+\frac{3h}{10+\sqrt{4-3h}} \right)}; h=\left( \frac{(1-E)}{(1+E)} \right)^{2}$ Eqn 4.

where *A* is the semi-major TT width (radius for a circle) and *E* the ratio of minor to major elliptical axes (using the Ramanujan approximation for an elliptical boundary).

To obtain *C_Cal_*/*C_FM_*_,_ we note that when the microscope focus is at the SS, the volume signal being measured (*SV_SS_*) should be half of that recorded when the microscope focus is in the bath because the PSF is bisected by the surface membrane, while the surface signal *SA_SS_* arises from a disk with whose area signal is equivalent to that of a plane bisecting the PSF. For a Gaussian PSF:

${SV}_{SS}=\frac{C_{Vol}}{2}\cdot\iiint_{-\infty}^{+\infty} e^{-\left( \frac{x^{2}}{{2\sigma}_{x}^{2}}+\frac{y^{2}}{{2\sigma}_{y}^{2}}+\frac{z^{2}}{{2\sigma}_{z}^{2}} \right)}dx dy dz$ Eqn. 5

and

${SA}_{SS}=C_{Area}\cdot\iint_{-\infty}^{+\infty} e^{-\left( \frac{y^{2}}{{2\sigma}_{y}^{2}}+\frac{z^{2}}{{2\sigma}_{z}^{2}} \right)}dy dz$ Eqn. 6

(In microscopy, the PSF is usually described by its full-width at half maximum (FWHM) which is related to σ by $FWHM=\sigma\cdot2\sqrt{2\ln2}$ ≈ 2.35σ).

Since the effective system PSF has been modified (by Gaussian blurring) to be symmetrical and therefore precisely known we can divide Eqns. 4 and 5 and integrate the result:

$\frac{{SV}_{ss}}{{SA}_{ss}}=\frac{C_{Vol}}{{2\cdot C}_{Area}}\int_{-\infty}^{+\infty} e^{-\left( \frac{x^{2}}{{2\sigma}_{x}^{2}} \right)} dx=\frac{C_{Vol}}{C_{Area}}\cdot\frac{FWHM}{4}\cdot\sqrt{\frac{\pi}{ln2}}$ Eqn. 7

Hence the ratio of the normalized volume signal to that of the normalized surface area signal in any sub-resolution circular cross-section TTs is:

$V:SA=\frac{r}{FWHM}\cdot\sqrt{\frac{ln2}{\pi}}$ Eqn. 8

Thus normalization of the intracellular signals to their surface and volume values and applying a spherical PSF produces a *V:SA* measure that depends only on local radius and the FWHM of the modified PSF (which, for our microscope, we set to 0.6μm). It should be noted that related calibration values can be derived for any arbitrary shape factor. This calibration was applied to all *V:SA* data presented here.
